# Short-term and medium-term clinical outcomes of laparoscopic-assisted and open surgery for colorectal cancer: a single center retrospective case-control study

**DOI:** 10.1186/1471-230X-11-85

**Published:** 2011-07-27

**Authors:** Jing Sun, Tao Jiang, Zhengjun Qiu, Gang Cen, Jun Cao, Kejian Huang, Ying Pu, Hong Liang, Renxiang Huang, Shifu Chen

**Affiliations:** 1 Department of General Surgery, Rui Jin Hospital, Shanghai Jiao Tong University School of Medicine, 197 Rui Jin Er Road, Shanghai, 200025, China; 2 Department of General Surgery, Affiliated First People's Hospital, Shanghai Jiao Tong University, 100 Hai Ning Road, Shanghai, 200080, China; 3 Department of Nursing, Affiliated First People's Hospital, Shanghai Jiao Tong University, 100 Hai Ning Road, Shanghai, 200080, China; 4 Department of General Surgery, Shanghai Minimally Invasive Surgery Center, 573 Xu Jia Hui Road, Shanghai, 200025, China

## Abstract

**Background:**

Laparoscopic procedure is a rapid developed technique in colorectal surgery. In this investigation we aim at assessing the diversities of short-term and medium-term clinical outcomes of laparoscopic-assisted versus open surgery for colorectal cancer.

**Methods:**

A total number of 519 patients with non-metastatic colorectal cancer were enrolled for this study. The patients underwent either laparoscopic-assisted surgery (LAP) (n = 254) or open surgery (OP) (n = 265). Surgical techniques, perioperative managements and clinical follow-ups were standardized. Short-term perioperative data and medium-term recurrence and survival were compared and analyzed between the two groups.

**Results:**

There were no differences in perioperative parameters between the two groups except in regards to a trend of faster recovery in laparoscopic procedures. There was no statistically significant difference in postoperative complications, reoperation rate, or perioperative mortality. Statistically significant differences in a faster return of gastrointestinal function and shorter hospital stay were identified in favor of laparoscopic-assisted resection. In colon and rectal cancer cases separately, the overall survival, cancer-free survival and recurrence rate were similar in two groups. There was also no tendency of significant differences in overall survival, cancer-free survival and recurrence in stage I-II and stage III patients in two cancer categories between the two groups, respectively. pT, lymph node metastasis, and clinical stage were independent predictors of overall death risk, while pT, pN, lymph node metastasis and clinical stage were found to be the independent predictors of recurrence risk in enrolled patients database.

**Conclusions:**

Laparoscopic-assisted procedure has more benefits on postoperative recovery, while has the same effects on medium-term recurrence and survival compared with open surgery in the treatment of non-metastatic colorectal cancer.

## Background

Colorectal cancer has gradually become one of the most significant leading causes of death from malignancies world-wide, especially in China. Surgical management is still the mainstay of the treatment [1,2]. Conventional open surgery is reported with significant morbidity and a long recovery period. With the laparoscopic techniques applied to the surgical field for colorectal diseases, laparoscopic colorectal surgery was first performed in Japan in 1992, very soon after its initial description by Jacobs et al [3,4]. In 1993, the first laparoscopic colectomy was successfully performed in China. Since then, laparoscopic surgery has been widely performed for various benign colorectal diseases [5-8], and furthermore, colorectal cancer.

Laparoscopic colorectal surgery is technically complicated as it involves almost all advanced laparoscopic techniques, such as mobilization, intracorporeal division, dissection of major vessels, and anastomosis. There is a steep learning curve to achieve advanced laparoscopic skills. But when the learning phase has been conquered, the benefits of laparoscopic surgery have been suggested with respect to decreased morbidity, decreased pain, faster recovery, shorter hospital stay and possibly reduced immunosuppression, comparing with open surgery [9-12].

However, behind the great success of laparoscopic colorectal surgery, there are still many questions that remain unclear, including whether laparoscopic colorectal cancer surgery is radical or not, seldom reported superior short-term outcomes. Laparoscopic colorectal surgery is still not considered standard treatment [13]. There are also controversies with potential port site recurrence after curative resection of tumor, not to mention too much economical costs.

In this article, we investigated the short-term and medium-term clinical outcomes of laparoscopic surgery versus open surgery for colorectal cancer over a period of 5 years in our center, aiming at investigating whether the laparoscopic surgery has any advantages for the patients with colorectal cancer or not.

## Methods

### Patient selection

Between January 2006 and June 2009, patients who underwent radical colorectal surgery for colorectal cancer in Affiliated First People's Hospital, Shanghai Jiao Tong University were consecutively enrolled in this study. Both open surgeries (OP) and laparoscopic-assisted surgeries (LAP) were performed by two stable surgical teams, respectively. No selection criteria were used to allocate patients to either a laparoscopic or an open operation. Patients were assigned to each surgical team (open or laparoscopic) according to their target dates for treatment and operating theatre availability. A minority of patients who wished to be operated laparoscopically were accommodated whenever possible. The trial received approval from local research ethics committee, and written informed consent was obtained from all patients before the investigation.

All patients enrolled accepted preoperative laboratory examination including tumor markers screening, coagulation test, chest x-ray, abdominal ultrasound, colonoscopy and if necessary, CT scan of the abdomen and pelvis. Endoscopic applications of metal clips for tumor localization, as well as intra-operative colonoscopic orientations were performed selectively. All patients were confirmed to have a malignant tumor after postoperative pathologic examination. Postoperative clinical staging after pathologic examination was based on the UICC cancer staging manual (2007). None of the patients had accepted preoperative radiotherapy or chemotherapy; out of the patients who were pathologically diagnosed as stage III, all accepted adjuvant chemotherapy with oxaliplatin and 5-fluorouracil for 6 months postoperatively. Exclusion criteria were: in situ or metastatic disease, emergency presentation, morbid obesity (defined as body mass index, i.e. BMI > 35 kg/m^2^), a classification V physical status according to the American Society of Anesthesiologists (ASA), associated gastrointestinal disease that required extensive operative evaluation or intervention, pregnancy or malignant disease in the past 5 years (except superficial squamous or basal cell carcinoma of the skin or in situ cervical cancer).

### Preoperative preparations and operation procedures

All patients had oral administration of gentamicin and metronidazole, 3 times a day for 3 days before surgery. Polyethylene glycol-electrolyte solution or magnesium sulfate was given one day before the surgery for bowel preparation. Other preoperative preparations were standardized, as followed for traditional abdominal surgeries.

Laparoscopic-assisted resection involved mobilization of the colon, visualization of critical structures, and intracorporeal vascular ligation. A standard total mesorectal excision (TME) procedure was essential for rectal cancer resection. A small abdominal incision was required to remove the specimen. Anastomosis was performed either through the small incision for right-hemi, transverse and left-hemi colon cancer, or laparoscopically with a double-stapling technique, for sigmoid and rectal cancer. If the tumor was located so close to the dentation line that anal-saving could not guarantee the radical standards and operation safety, the Hartmann & Bacon procedures were performed. In the majority of cases, the operation was performed utilizing a lateral to medial approach. In this study, an incision longer or different to that planned was used to determine a conversion. Conversion to open colectomy was at the discretion of the surgeon based on concerns regarding patient safety, technical difficulties, or associated unexpected conditions requiring treatment by laparotomy. Conversions were recorded and analyzed as part of the laparoscopic arm of the study, but were excluded for further analysis. Open procedures were performed according to the standard techniques followed by the operating surgeon. All operations achieved a standard D2 lymph node dissection according to the Guidelines of Radical Laparoscopic Colorectal Cancer Surgery (2006, 2008) established by the Study Group of Laparoscopic and Endoscopic Surgery Affiliated to Chinese Medical Association, as well as the General Rules for Clinical and Pathological Studies on Cancer of the Colon, Rectum and Anus, 5^th ^edition (1994), by the Japanese Research Society for Cancer of the Colon and Rectum.

### Perioperative surveillance, postoperative managements and follow-up evaluation

Demographic and operative data were obtained regarding age, gender, BMI, ASA score, comorbidities, history of previous abdominal surgery, tumor location, surgical intervention, operative time, blood loss, maximum incision length, sample length, proximal and distal margin length, number of retrieved lymph nodes and lymph node metastases, tumor size, pathological differentiation and clinical stage. Postoperative data included analgesic usage, Visual Analog Scales (VAS) score, peristalsis recovery time, time until flatus, time until off-bed, time until first liquid and semi-liquid intake, postoperative duration of hospital stay and total time of hospital stay, were recorded.

Patients enrolled in the present study were managed postoperatively by the same group of surgeons. Patients in both groups were supported by infusions in the very first several hours after surgery. After confirmation of the peristalsis recovery, liquid diet was supplied. Semi-liquid diet was considered suitable for patients after report of flatus. For pain control, patients were given patient-controlled anesthesia (PCA) or short-acting drugs according to their own aspirations. Prophylactic antibiotics were used during 72 hours after surgery; however, if there was any indication of infection, this time was prolonged. The catheter was removed as early as possible except for patients with tumors located in the lower region of the rectum. The peritoneal drain was removed on POD7, only if no leakage or hemorrhage was confirmed, as well as the patient had taken semi-liquid food and had reported a formed stool. In patients with postoperative complications, the management was almost the same in both treatments groups, respectively. All patients were followed-up after being discharged from the hospital, according to a pre-established protocol. This included recording of medical history, physical examination, and laboratory studies such as, serum carcinoembryonic antigen (CEA) and carbohydrate antigen 19-9 (CE19-9) levels, which were assessed 1 month after surgery and every 3 months thereafter. At each patient visit, symptoms were recorded and wound scars examined for subcutaneous metastasis. Either ultrasonography or CT scan of the abdomen, in addition to chest X-ray was performed every 6 months whereas total colonoscopy was performed every year. When colonoscopy was incomplete, a combination of sigmoidoscopy and barium enema methods was undertaken. Recurrences were histologically confirmed and classified as distant metastasis, locoregional relapse (tumor growth restricted to the anastomosis or the region of primary operation), and incision/port-site metastasis. The last date for follow-ups was March, 2011.

### Statistical analysis

Data were collected prospectively using a computerized data base according to pre-study Power calculation. Quantitative data was given as a mean ± standard deviation, and analyzed using Student's t-test. Count data between LAP and OP groups was assessed by Mann-Whitney, Chi-Square or Fisher's exact test as appropriate. Comparisons between the two groups were made on an intention-to-treat basis; thus, patients in the LAP group converted to the open procedure were not excluded from the analysis. Time to: (1) last follow-up evaluation, (2) treatment failure or (3) death was measured from the date of operation.

Recurrence and overall survivals were evaluated using the Kaplan-Meier method and compared with the log-rank test. Analysis of predictive factors of survival was performed. Variables analyzed univariately were, age, gender, BMI, ASA scores, preoperative comorbidities, tumor location, intervention, surgical procedures, tumor size, pT, pN, pathological differentiation, lymph nodes metastasis, clinical stage, and postoperative complications. Variables associated with recurrence or survival, were then used for multivariate analysis using a stepwise Cox proportional-hazards regression model. Statistical significance was defined as P < 0.05. All calculations were performed by using the SPSS software package version 12.0 (SPSS Inc., Chicago, IL).

## Results

A total of 519 patients were enrolled and analyzed in this trial: 254 underwent laparoscopic-assisted colorectal resection and 265 were treated with open colorectal resection. There was no statistically significant difference found in the majority of the demographic parameters between the two patient populations (Table 1).

**Table 1:** Demographic Data and Intraoperative Data

		LAP (n = 254)		OP (n = 265)		P
		n	%	n	%	
Age (Years, mean ± SD)		67.5 ± 10.8		66.0 ± 12.1		0.137
Gender	Male	138	54.3	126	47.5	0.136
	Female	116	45.7	139	52.5	
BMI (kg/m^2^, mean ± SD)		23.4 ± 4.1		23.5 ± 3.9		0.622
ASA Score	1	17	6.7	27	10.2	0.222
	2	148	58.3	160	60.4	
	3	83	32.7	69	26.1	
	4	6	2.3	9	3.3	
Abdominal Operation History						1.000
	No	194	76.4	202	76.2	
	Yes	60	23.6	63	23.8	
Preoperative Comorbid Diseases						0.333
	No	109	42.9	125	47.2	
	Yes	145	57.1	140	52.8	
	Cardiovascular	113		111		
	Respiratory	10		11		
	Hepatic Cirrhosis	0		1		
	Renal Failure	4		6		
	Cerebral Infarction	7		8		
	Diabetes	39		30		
	Autoimmunal	1		1		
	Others	17		15		
Intervention	Right Colectomy	70	27.6	72	27.2	0.906
	Transverse Colectomy	3	1.2	6	2.2	
	Left Colectomy	16	6.2	15	5.7	
	Sigmoidectomy	67	26.4	68	25.7	
	HAR	51	20.1	56	21.1	
	LAR	17	6.7	26	9.8	
	APR	27	10.6	19	7.2	
	Hartmann	1	0.4	3	1.1	
	Bacon	2	0.8	0	0.0	
Conversions to Open^a^		8	3	/	/	
Operative Time (min, mean ± SD)		135.6 ± 40.0		131.2 ± 46.0		0.307
Blood Loss (ml, mean ± SD)		101.5 ± 70.8		104.7 ± 118.8		0.002
Maximum Incision (cm, mean ± SD)		4.3 ± 1.1		13.6 ± 2.3		0.000

BMI, Body Mass Index; ASA, American Society of Anesthesiologists; HAR, High Anterior Resection; LAR, Low Anterior Resection; APR, Abdomioperineal Resection; a, 1 HAR, 1 LAR, 1 Sigmoid Colostomy, 2 Right Colectomy, and 3 Sigmoidectomy; All conversions on account of severe local invasion and adhesion (Excluded from Laparoscopic group)

### Operative Parameters

There was no statistically significant difference in the operative time between the groups. Furthermore, we observed decreased blood loss during laparoscopic surgery, which was statistically significant (P value?). Patients in the LAP group had significantly shorter surgical incisions (P = 0.000). Eight cases (3.0%) were converted from laparoscopic to open surgery. Reasons for conversion were severe local invasion (2 cases, 0.8%) and adhesion (6 cases, 2.3%) (Table 1).

### Pathological Evaluation

The majority of tumors were located in the rectum, right-hemi colon, and sigmoid colon, which is consistent with the colorectal epidemiology scenario in China, with no differences between two groups (P = 0.896). The predominant tumor type was adenocarcinoma with the majority being moderately differentiated in both groups (P = 0.222) (Table 2).

**Table 2:** Pathological Parameters

		LAP (n = 254)		OP (n = 265)		P
		n	%	n	%	
Location	Right-hemi Colon	70	27.6	72	27.2	0.896
	Transverse Colon	3	1.2	6	2.2	
	Left-hemi Colon	16	6.2	15	5.7	
	Sigmoid Colon	65	25.6	68	25.7	
	Rectum	100	39.4	104	39.2	
Tumor Size (cm, mean ± SD)		4.7 ± 1.7		4.8 ± 1.9		0.579
Proximal Margin for colon cancer (cm)^a^		12.7 ± 6.8		11.4 ± 4.1		0.000
Distal Margin for colon cancer (cm)^a^		10.7 ± 6.7		9.8 ± 5.7		0.189
Proximal Margin for rectum cancer (cm)^b^		11.4 ± 4.3		11.7 ± 4.7		0.573
Distal Margin f for rectum cancer(cm)^b^		4.8 ± 3.1		4.0 ± 1.7		0.052
Total Sample Length(cm)		26.3 ± 10.5		25.4 ± 8.9		0.302
Lymph Nodes Retrieved		12.0 ± 6.9		11.4 ± 7.1		0.327
Pathological Results						
	Differentiation					0.222
	Well-differentiated	51	20.1	53	20.0	
	Moderate-differentiated	172	67.7	166	62.6	
	Poor-differentiated	16	6.3	17	6.5	
	Mucinous	15	5.9	29	10.9	
	pT					0.362
	pT1	33	13.0	27	10.2	
	pT2	63	24.8	52	19.6	
	pT3	75	29.5	107	40.4	
	pT4	83	32.7	79	29.8	
	pN					0.661
	pN0	104	40.9	117	44.2	
	pN1	83	32.7	81	30.6	
	pN2	67	26.4	67	25.3	
	Lymph Nodes Metastasis					0.478
	Yes	150	59.1	148	55.8	
	No	104	40.9	117	44.2	
	TMN Stage					0.286
	I	26	10.2	17	6.4	
	II	103	40.6	111	41.9	
	III	125	49.2	137	51.6	

^a^Laparoscopy group n = 154, open group n = 161.

^b^Laparoscopy group n = 100, open group n = 104.

Resection margins were measured in fresh specimens after surgery, without fixation with formalin (Table 2). The proximal margin in colon cancer group were 12.7 ± 6.8 cm in LAP and 11.4 ± 4.1 cm in OP group respectively (P = 0.000). The distal margin in rectal cancer group were 4.8 ± 3.1 cm in LAP and 4.0 ± 1.7 cm in OP group (P = 0.052), respectively. The postoperative pathological confirmation indicated that there was no case of positive resection margins. Thus, no patient was required to return for a further resection to ensure adequate margins were obtained. Fresh resection lengths were 26.3 ± 10.5 cm in the LAP group and 25.4 ± 8.9 cm in the OP group (P = 0.302).

The lymph nodes were retrieved from each sample after fixation, by the standby surgeon and pathologist and reconfirmed to be metastatic using immunohistochemistry. The lymph nodes retrieved from the LAP group were 12.0 ± 6.9, while from the group treated with open surgery were 11.4 ± 7.1 (P = 0.327). There were no significant differences in pT, pN, lymph node metastasis or overall TNM staging (P = 0.362, 0.661, P = 0.478, P = 0.286) (Table 2).

### Perioperative Complications and Reoperations

No significant difference was found in the number of adverse events during the operation procedures (Table 3). Of the adverse events that occurred during laparoscopic procedures, there was: 1 massive hemorrhage (approximately 1500 ml, requiring blood transfusion during the surgery), 1 left ureter injury during sigmoidectomy, 1 duodenum injury during right colectomy, 2 instrument disorders, and 1 subcutaneous emphysema. During the open surgery, 1 massive hemorrhage (approximately 1000 ml, requiring blood transfusion during the surgery), 1 left ureter injury during HAR (High Anterior Resection) and 1 stapler-failure causing leakage during LAR (Low Anterior Resection) were reported.

**Table 3:** Perioperative Complications for Colorectal Cancer

	LAP (n = 254)		OP (n = 265)		P
	n	%	n	%	
Intraoperative Complications	6	2.4	3	1.1	0.330
Massive Hemorrhage (> 1000 ml)	1		1		
Organ Injury	2		1		
Equipment Disorders	2		0		
Others	1^a^		1^b^		
Post-operative Complications	33	12.9	34	12.8	0.530
Ileus	6		5		
Anastomotic Hemorrhage	1		0		
Abdominal Hemorrhage	1		1		
Peritonitis/Septic Shock	1		1		
Anastomotic Leakage	5		4		
Pelvic Abscess	2		3		
Wound Infection	5		6		
Incisional/Port Herniation	3		2		
Cardiovascular Disorders	1		3		
Respiratory Disorders	0		3		
Renal Failure	0		2		
Urinary Infection	2		1		
Cerebral Disorders	1		1		
Colostomy Stoma	1		0		
Others	4^c^		2^d^		
Re-operative cases	7^e^	2.8	3^f^	1.1	0.213

^a ^1 of Subcutaneous Emphysema.

^b ^1 of stapler leakage during LAR.

^c ^1 of Lymphatic Fistula, 1 of Deep Vein Thrombosis, 1 of Common Peroneal Nerve Injury, 1 of Pneumoperitoneum-related Acidosis.

^d ^1 of Urinary Retention, 1 of Recto-vaginal Fistula.

^e ^1 Re-colostomy for Colostomy Stoma Necrosis, 1 Hartmann for Anastomotic Hemorrhage, 1 Laparotomy for Anastomotic Obstruction, 1 Hartmann for Leakage, 3 Transverse Colostomies for Leakage.

^f ^1 Abscess Drainage for Pelvic-perianal Abscess, 1 Transverse Colostomy for Leakage, 1 Reconstruction for Recto-vaginal Fistula.

The number of patients with at least 1 intraoperative complication was not significantly greater among the laparoscopically treated patients as expected: 6 (2.4%) versus 3 (1.1%) (P = 0.330). Moreover, the number of patients with at least 1 postoperative complication was not significantly different between the 2 groups: 33 (12.9%) for laparoscopic surgery versus 34 (12.8%) for open surgery (P = 0.530). All cases of anastomotic leakage occurred only in LAR patients in both groups. Among the laparoscopically treated group, one each of 4 rare complications: lymphatic fistula, deep vein thrombosis, common peroneal nerve injury and pneumoperitoneum-related acidosis, were seen. These were the typical adverse events of laparoscopic surgery. In the OP group one case each of urinary retention and recto-vaginal fistula were reported.

There was no statistically significant difference in the rate of reoperation between the 2 groups: 7 (2.8%) for laparoscopic surgery versus 3 (1.1%) for open surgery (P = 0.213). There were 4 (1.4%) anastomotic leaks in the LAP group with reoperation compared with a single case (3.4%) reported from the OP group (P = 0.173). Four reoperation cases among laparoscopically treated patients included 1 Hartmann and 3 transverse colostomies, compared to 1 case of transverse colostomy requiring reoperation in the OP group. Three additional reoperations among LAP treated patients were: re-colostomy for colostomy stoma necrosis, Hartmann for anastomotic hemorrhage, laparotomy for anastomotic obstruction. Two further reoperations in the patient group who received open surgery treatment were associated with abscess drainage for pelvic-perianal abscess and reconstruction for recto-vaginal fistula.

### Perioperative Recovery and Mortality

The patients in the LAP group showed statistically significant faster recovery than the OP group. 66.1% (168/254) of patients in the LAP group did not need analgesic for pain-control after surgery, and only 20.5% (52/254) of patients need PCIA (patient controlled intravenous analgesia) for pain-control. Most of the patients in the OP group demanded PCIA for after-surgery pain-control as a requisite treatment, and only 19.6% (52/265) of patients could pass the recovery period peacefully without any analgesic. Laparoscopic colorectal surgery obviously caused significantly less pain for patients (P = 0.008). The average time after which the patient got off from bed was 2.8 ± 1.8 days for LAP group versus 4.7 ± 3.1 days for OP group (P = 0.000). Peristalsis recovery, flatus time, postoperative hospital stay, and total hospital stay were all significantly shorter in the LAP group (P = 0.000). Regarding time to oral intake, although the results showed a significantly earlier time among the laparoscopically treated patients, actually this time was found to be influenced by surgeon's experiences and customs to a great extent. Since the two groups were managed by different surgical teams respectively, the variation in oral intake time had to be excluded from the evaluation of postoperative recovery (Table 4).

**Table 4:** Postoperative Recovery

	LAP (n = 254)		OP(n = 265)		P
	n	%	n	%	
Analgesic Usage					0.000
No	168	66.1	52	19.6	
Short-acting Drug	34	13.4	18	6.8	
PCIA	52	20.5	195	73.6	
VAS Score (NRS) on POD#1 (mean ± SD)	2.8 ± 1.6		3.2 ± 1.9		0.008
Off-bed (day, mean ± SD)	2.8 ± 1.8		4.7 ± 3.1		0.000
Peristalsis Recovery (day, mean ± SD)	1.8 ± 0.8		2.5 ± 1.2		0.000
Flatus (day, mean ± SD)	3.2 ± 1.6		4.3 ± 2.9		0.000
Liquid Intake (day, mean ± SD)	3.6 ± 2.1		5.3 ± 4.3		0.000
Semi-liquid Intake (day, mean ± SD)	6.1 ± 2.5		8.2 ± 4.7		0.000
Post-op Hospital Stay (day, mean ± SD)	9.8 ± 4.7		13.1 ± .7.2		0.000
Total Hospital Stay (day, mean ± SD)	17.6 ± 7.4		22.9 ± 9.3		0.000

PCIA, Patient Controlled Intravenous Analgesia, personalized prescription of tramadol and fentanyl. VAS, Visual Analog Scales; NRS, Numerical Rating Scales.

There were 2 cases of perioperative mortality in each group, which was excluded from the follow-up investigation (Table 5). One laparoscopically treated patient died due to complications arising from an anastomotic leak, diagnosed as septic shock, which developed into multiple organ disorder syndrome (MODS) leading to death on POD#19. A second death recorded, was due to a sudden cardiac arrest on POD#1. In the OP group, both mortalities, on POD#6 and POD#7 respectively, were reported to have cardiovascular causes.

**Table 5:** Postoperative Recurrence and Survival for Colorectal Cancer

	Colon Cancer				P	Rectal Cancer				P
	LAP (n = 154)		OP(n = 161)			LAP (n = 100)		OP(n = 104)		
	n	%	n	%		n	%	n	%	
Overall Mortality	33	21.4	45	28.0	0.113	25	25.0	29	27.9	0.439
Cause of Death										
Peri-operative Mortality	1^a^		1^c^			1^b^		1^c^		
Tumor Progression	31		42			22		27		
Others	1^d^		2^e^			2^f^		1^g^		
Tumor Recurrence	44	28.6	47	29.2	0.501	33	33.0	32	30.8	0.482
Type of Recurrence										
Locoregional Relapse	18		21			17		18		
Metastasis	24		25			15		14		
Incision/Port Metastasis	2		1			1		0		

^a ^1 on POD#19 for septic shock (Excluded from Laparoscopic group for survival analysis).

^b ^1on POD#1 for cardiac arrest (Excluded from Laparoscopic group for survival analysis).

^c ^2 on POD#6, POD#7 respectively for cardiovascular accident (Excluded from Open group).

^d ^1 for traffic accident.

^e ^1 for unknown reason, 1 for cardiac infarction.

^f ^1for cerebral hemorrhage, 1 for cardiac infarction.

^g ^1 for cardiac infarction.

### Follow-up Recurrence and Survival

A 100% patient follow-up was achieved in the present study. All patients were compliant with the proposed postoperative surveillance protocol. The total follow-up duration was from 8.2 to 62.5 months in the whole series (8.2 to 62.5 months in the LAP group, and 10.4 to 62.5 months in the OP group). The median follow-up times were 37.1 months and 36.7 months in the laparoscopically operated and open surgically treated groups, respectively.

In both colon and rectal cancer cases, there was no difference in the overall mortality trend between the two treatment groups (P = 0.113, 0.439). The number of deaths, respectively, in the laparoscopically and open surgically treated groups, among colon cancer cases was 33 patients (21.4%) versus 45 patients (28.0%), while among rectal cancer cases was 25 patients (25.0%) versus 29 patients (27.9%) (Table 5).

**Figure 1:**
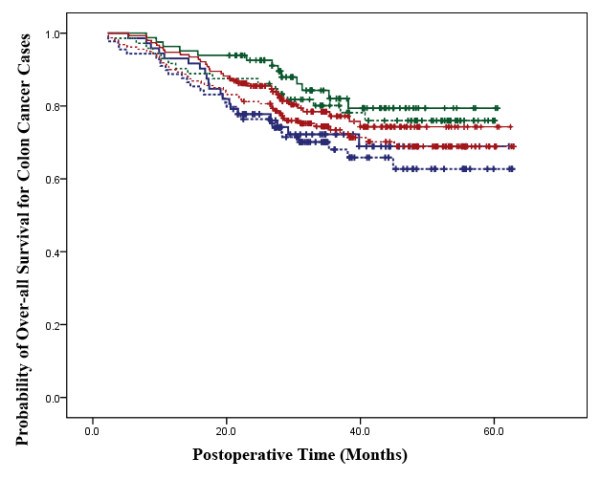
**Survival Analysis in Colon Cancer Cases**. The laparoscopic-assisted group is represented by a continuous line and the open surgery group is represented by a dotted line. The red lines represent overall survival for all stages in the two groups (P = 0.305). The green lines represent overall survival for stage I-II patients in the two groups (P = 0.498). The blue lines represent overall survival for stage III patients in the two groups (P = 0.629).

**Figure 2:**
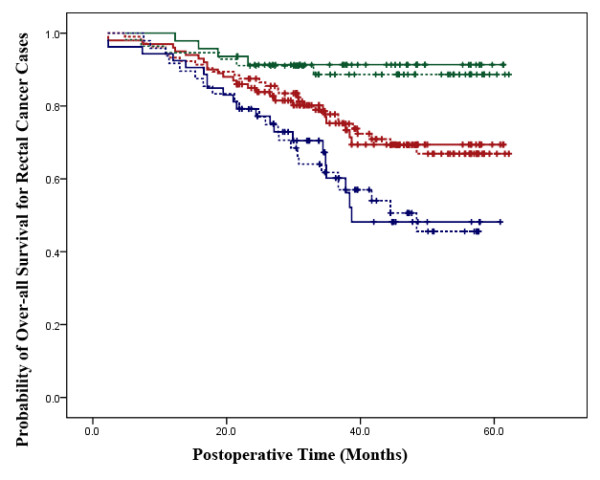
**Survival Analysis in Rectal Cancer Cases**. The laparoscopic-assisted group is represented by a continuous line and the open surgery group is represented by a dotted line. The red lines represent overall survival for all stages in the two groups (P = 0.954). The green lines represent overall survival for stage I-II patients in the two groups (P = 0.723). The blue lines represent overall survival for stage III patients in the two groups (P = 0.949).

According to the results of Kaplan-Meier analysis, in colon cancer cases, the two treatment groups did not have significant differences in overall survival (P = 0.305). When patients were stratified according to the tumor stage, the probabilities of overall survival were not significantly different between the two treatment groups for stage I-II (P = 0.498) and stage III (P = 0.629) tumors (Figure 1). Consistent to our observation in the colon cancer cases, among the rectal cancer patients, the two treatment groups did not have significant differences in overall survival (P = 0.954). When patients were stratified according to the tumor stage, the probabilities of overall survivals were not significantly different in the LAP group compared with OP group for stage I-II (P = 0.723) as well as stage III (P = 0.949) tumors (Figure 2).

**Figure 3:**
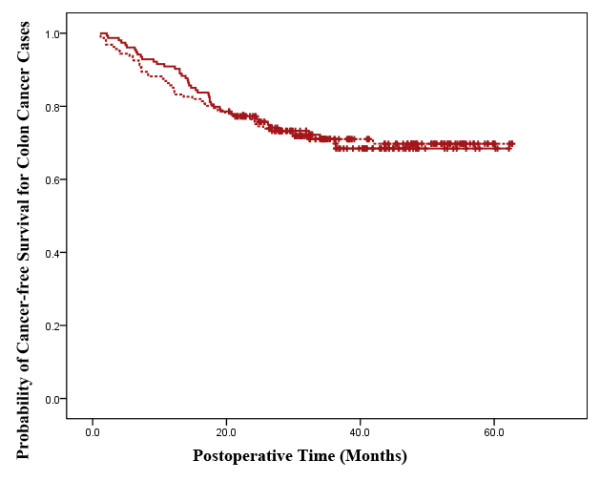
**Cancer-free Survival Analysis in Colon Cancer Cases**. The laparoscopic-assisted group is represented by a continuous line and the open surgery group is represented by a dotted line. The cancer-free survival for all colon cancer cases in the two groups have no significant difference (P = 0.973).

**Figure 4:**
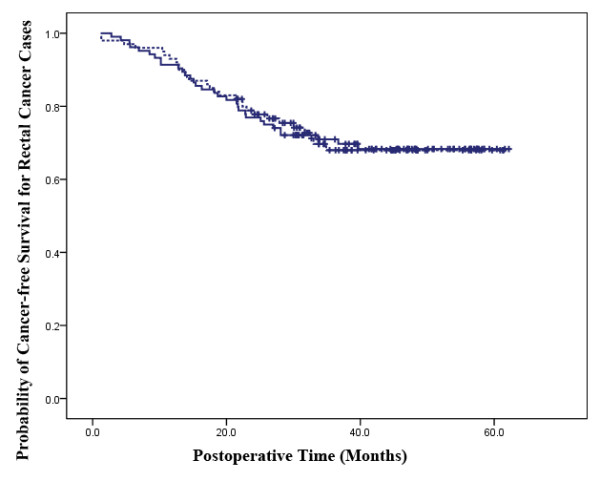
**Cancer-free Survival Analysis in Rectal Cancer Cases**. The laparoscopic-assisted group is represented by a continuous line and the open surgery group is represented by a dotted line. The cancer-free survival for all rectal cancer cases in the two groups have no significant difference (P = 0.968).

In colon and rectal cancer cases, the tumor recurrence rates tended to be similar in both treatment groups (P = 0.501, 0.482). In the colon cancer cohort, 44 patients (28.6%) from the LAP group and 47 patients (29.2%) from the OP group showed tumor recurrence. Tumor recurrence was reported in 33 (33.0%) and 32 rectal cancer patients (30.8%), respectively, from the laparoscopically and OP groups (Table 5). The overall cancer-free survival duration according to Kaplan-Meier analysis also showed similar results in the two cancer categories, respectively (P = 0.973, 0.968) (Figures 3 and 4).

### Prognostic Analyses

As indicated by the univariate analysis, pN, lymph node metastasis, and clinical stage (P = 0.001, P = 0.000, P = 0.000, respectively) were found to be independent predictors of overall death risk, while tumor diameter, pT, pN, lymph node metastasis and clinical stage (P = 0.044, P = 0.008, P = 0.008, P = 0.002, P = 0.000, respectively) were found to be independent predictors of recurrence risk (Table 6).

**Table 6:** Univariate Risk Evaluations for Recurrence and Survival

Factors	Over-all Death Risk (n = 519)		Recurrence Risk (n = 519)	
	Chi^2^	P	Chi^2^	P
Gender	0.847	0.357	0.270	0.604
Age (years,≤65, > 65)	2.642	0.104	2.398	0.122
BMI (kg/m^2^, ≤24, > 24)	0.451	0.502	0.128	0.721
ASA Score	1.675	0.642	2.996	0.392
Pre-op Comorbidities	2.460	0.117	1.672	0.196
Tumor Location	8.726	0.068	6.570	0.160
Intervention	12.510	0.085	8.843	0.264
Surgical Procedures	0.588	0.443	0.002	0.968
Tumor Diameter (cm, ≤5, > 5)	2.279	0.133	4.040	0.044
pT	1.616	0.656	11.776	0.008
pN	14.476	0.001	9.615	0.008
Pathological Differentiation	1.565	0.667	1.808	0.613
Lymph Node Metastasis	14.714	0.000	9.811	0.002
Clinical Stage	32.953	0.000	24.848	0.000
Post-op Complications	0.689	0.407	0.220	0.639

However, the multivariate analysis indicated that pT, lymph node metastasis, and clinical stage (P = 0.049, P = 0.003, P = 0.000, respectively) were all independent predictors of overall death risk, while pT, pN, lymph node metastasis and clinical stage (P = 0.023, P = 0.015, P = 0.005, P = 0.000, respectively) were found to be the independent predictors of recurrence risk (Table 7).

**Table 7:** Multivariable Risk Evaluation for Recurrence and Survival

Factors	Over-all Death Risk (n = 519)			Recurrence Risk (n = 519)		
	Chi^2^	HR (95%CI)	P	Chi^2^	HR (95%CI)	P
Gender	-0.236	0.790 (0.554-1.125)	0.191	-0.136	0.873 (0.629-1.213)	0.418
Age	0.446	1.813 (0.976-4.045)	0.047	0.216	1.241 (0.883-1.745)	0.213
BMI	-0.243	0.784 (0.549-1.120)	0.181	-0.125	0.882 (0.635-1.227)	0.456
ASA Score	0.236	1.267 (0.965-1.663)	0.089	0.091	1.095 (0.851-1.409)	0.481
Pre-op Comorbidities	0.173	1.189 (0.822-1.720)	0.359	0.107	1.113 (0.791-1.565)	0.540
Tumor Location	-0.088	0.916 (0.809-1.036)	0.161	-0.062	0.939 (0.840-1.051)	0.275
Intervention	-0.049	0.952 (0.871-1.041)	0.279	-0.030	0.971 (0.893-1.055)	0.485
Surgical Procedures	0.175	1.192 (0.833-1.706)	0.338	0.018	1.018 (0.732-1.416)	0.914
Tumor Diameter	0.212	1.236 (0.858-1.781)	0.255	0.280	1.324 (0.944-1.856)	0.104
pT	0.536	1.965 (0.807-12.153)	0.049	0.632	1.533 (0.874-10.220)	0.023
pN	0.513	1.713 (0.647-11.584)	0.056	0.576	1.913 (0.665-13.544)	0.015
Pathological Differentiation	-0.172	0.842 (0.659-1.077)	0.171	-0.154	0.857 (0.683-1.075)	0.182
Lymph Node Metastasis	0.763	9.757 (2.186-43.546)	0.003	8.280	20.074 (2.602-154.853)	0.005
Clinical Stage	1.354	3.875 (2.343-6.407)	0.000	1.031	2.803 (1.788-4.396)	0.000
Post-op Complications	-0.309	0.734 (0.386-1.395)	0.345	-0.173	0.841 (0.476-1.488)	0.552

95% CI, 95% Confidence Interval

## Discussion

We report the Chinese experience of performing laparoscopic-assisted colectomy for colorectal cancer. We found that LAP for colorectal cancer is feasible and safe; patients had acceptable rates of complications and conversion to open laparotomy, as well as reasonably short postoperative durations of stay, a large number of lymph node retrieval, and finally, similar survival rates.

With the development of laparoscopic techniques, along with the improvement of laparoscopic instruments, a standard laparoscopic procedure for colorectal cancer surgery has gradually become widely accepted, and a radical cure resection seems feasible for laparoscopic surgeries. The present study showed that there were no differences in the outcomes between the two treatment groups. Apart from acquirement of a new skill the laparoscopic surgeon being a factor, which cannot be totally ignored, this was a straightforward comparison. Furthermore, there was no apparent deterioration in the quality of surgery associated with the introduction of laparoscopic resection, as stoma formation and APR rates in rectal cancer remained unchanged over time. The intraoperative comparison between the two groups in our study indicated almost similar operative time and complications, which was not in keeping with other randomized controlled studies [14-20]. The mean operating time for the laparoscopic-assisted procedure was shorter in this study than in the Multicentre Randomized Controlled trial - Conventional versus Laparoscopic-Assisted Surgery In patients with Colorectal cancer (MRC CLASICC) [14] trial but similar to the Colon Carcinoma Laparoscopic or Open Resection (COLOR) [15,17] trial.

The number of lymph nodes harvested during the surgical procedure influences clinical staging of the tumor and is not only influenced by the operative technique or the extent of lymphadenectomy, but to an even greater extent by pathological techniques involved in processing the specimens. Examining fewer than 12 lymph nodes in a specimen can result in under-staging [21]. Since the specimens retrieved by either laparoscopic-assisted or open resection were processed in different ways, it has been difficult to compare the harvested lymph nodes in different studies. Nevertheless, since the standard D2 lymph node dissection was consistently followed for all operations, and the lymph node was always collected by a permanent surgeon and a permanent pathologist, a diminished bias during lymph node collection was assured. Our final analysis confirmed that there were no differences in lymph nodes harvested between the LAP and OP groups in this study, with the majority of patients having sufficient lymph nodes to be collected for accurate staging.

In previous reports with data on resection margins, none of the margins was found to be positive. Although this is a remarkable finding, it can be explained by the fact that most of these studies [22-27] only reported distal and proximal margins. No data on circumferential margins were available from these studies. Results of the primary analysis indicated that laparoscopic procedure might have the ability to reach a better dissect field than the open procedure, assuring the radical cure resection.

Among our patients, those who underwent laparoscopic procedure had significantly faster recovery than those who underwent open surgery. LAP group patients definitely need a smaller dose of analgesic than their counterparts who received open surgery treatment. In fact, most laparoscopic procedures seem to cause less pain, so that analgesics are rarely necessary. It is reported that some centers are in favor of the epidural combined with general anesthesia during the operation. Thus, usually the PCEA (patient controlled epidural analgesia) and PCIA are both usual options of postoperative pain-control procedures. Some reports concluded that PCEA has greater advantages over PCIA [28,29]. In our center, general anesthesia is used routinely for laparoscopic surgery; PCIA is the choice only for patient controlled pain-control procedures. However, since the majority of LAP group patients did not require analgesia, the pain-control method did not seem to be an important parameter for laparoscopic colorectal surgery.

Total hospital stay and postoperative hospital stay are two important evaluation criteria for fast-recovery surgery. The postoperative hospital stay for LAP and OP group in the Multicentre Randomized Controlled trial - MRC-CLASICC Trial was 9 days and 11 days, respectively [14]; however, the Clinical Outcomes of Surgical Therapy Study group (COST) Trial was 5.1 days and 5.6 days, respectively [22,23]. Length of hospital stay is an indicator which can be easily affected by different confounding factors, such as geographic locations, reflecting cultural and possibly financial reimbursement differences [30]. In our group, all stage III patients accepted postoperative adjuvant chemotherapy. There was a set of patients in both groups who could not be discharged until the end of the first regimen of chemotherapy. This undoubtedly extended the length of hospital stay for these patients, thus introducing bias in the comparison of hospital stay between the two groups. Thus, we calculated the actual hospital stay after eliminating any such excess periods of stay during the investigation.

The anastamotic leakage rate in LAR patients is significantly higher in laparoscopically treated cases (5/17, 29%) than in the OP group (4/26, 15%). However, after revisiting the data in the LAP group we discovered that all the leakage occurred in early cases; this may be explained as effect of learning curve. The investigation enrolled these patients when our laparoscopic surgeon was in the early stages of learning curve, which led to a higher rate of complications. However, for the open surgery, all enrolled patients were operated by a surgeon with experience of more than 500 cases of open colorectal cancer surgeries.

The conversion rate in this study was only 3%, which was far lower than that reported in other trials. The conversion rate from laparoscopic to open surgery was 17% in the COLOR trial [15,17], 25.4% in the COST [22] and 29% in the MRC-CLASICC [14] trial. The MRC-CLASICC trial included cases of both rectal cancer and advanced stage cancer; 34% of patients with cancer of the rectum compared with 25% of patients with cancer of the colon underwent conversion from laparoscopic to open procedure. The ALCCaS trial did not include patients with rectal cancer [31]. It is also worth noting that in the MRC-CLASICC trial the rate of intraoperative conversions fell by the year of study from 38% in the first year to 16% in the sixth year [14]. In our study, the laparoscopic procedures were all performed by a single surgeon and the conversion cases all reported in the early period. However, as time passed the experience in the procedure increased. With stabilization of the learning curve of the operating surgeon, the conversion rate significantly reduced. Furthermore, in our study stage IV patients were not included, and all patients were found in preoperative evaluation to be suitable for laparoscopic procedure, thus the conversion rate was lower than other trials. It was reported that there was no difference when comparing conversions to those completed in operative time, morbidity, length of stay, costs, and readmission [32]. There was greater blood loss, longer time to first bowel movement, longer length of stay when converted cases were compared with the cases completed with the laparoscopic-assisted approach but no difference when compared with open surgery [33].

Additionally, our study showed similar overall recurrence rate, as well as the survival rate between the two groups. The number of patients that developed a recurrence at the site of the primary tumor during the follow-up period of the study was similar to that after laparoscopic and open surgery in other trials respectively. Separate analyses for colon and rectal cancer showed no significant differences between laparoscopic and open procedures. No significant differences in the occurrence of port-site/wound metastases or peritoneal metastases were observed [22,34-39]. Long-term outcome data from three major multicenter trials are still awaited [15,22,35]. Early results from the COST study, with a median follow-up of 4.4 years, also did not demonstrate any difference in tumor recurrence, disease-free survival, and overall survival rates between the two surgical techniques for treating potentially curable colon cancer [22]. This study also confirmed that the laparoscopic approach can provide as good radical resection as an open approach for treating potentially curative colorectal cancer. Similar perioperative mortality in the two groups confirmed the clinical safety of the laparoscopic approach reported in earlier research [14,22,34,40]. The potential impact of laparoscopic surgery on survival is not clear in this study since the multivariance analysis only indicated the clinical staging as a high risk factor for overall survival. The role of immunosuppression has been proved because of mediators of immunologic response. This positive impact of the laparoscopic procedure is probably worth further investigation.

The present study was obviously limited in that the patients were partially randomized into the two treatments arms. However, these results were obtained by two different teams specializing in respective surgical procedures, operating at a high volume of cases. Moreover, since there were no differences in demographic data, and all observed biases have negligible impact on the results we believe our results are accurate. This study has confirmed the feasibility of laparoscopic procedures for colorectal cancer, advocating the fast recovery times, and demonstrating similar medium-term recurrence and survival between LAP and OP groups. Thus, in a dedicated laparoscopic center, laparoscopic procedures may result in a potential perioperative and follow-up survival benefit compared with open procedures, particularly in advanced cases.

## Conclusions

In this original clinical research, we conclude that laparoscopic-assisted procedures have more benefits on postoperative recovery, while has the same effectiveness on medium-term recurrence and survival compared with open surgery in the treatment of non-metastatic colorectal cancer. Thus, laparoscopic procedures may become the most effective treatments for colorectal cancer in the future.

## Competing interests

The authors declare that they have no competing interests.

## Authors' contributions

SJ, JT and QZ conceived the study, and participated in its design and coordination as well as drafting of the manuscript. CG, CJ, HK and CS carried out the perioperative management for all patients in this study, participated in the operation and contributed to the drafting of the manuscript. PY managed the equipment and instruments for the surgery as well as the intraoperative data collection. LH and HR participated in conducting the follow-up investigations. All authors have read and approved the final manuscript.

## Authors' information

The first author, Sun Jing MD was working in the Department of General Surgery of Shanghai Jiaotong University Affiliated First People's Hospital during this study, until September 2010. Currently he is enrolled in a PhD study program in the Department of General Surgery of Shanghai Ruijin Hospital and Shanghai Minimally Invasive Surgery Center.

## Pre-publication history

The pre-publication history for this paper can be accessed here:

http://www.biomedcentral.com/1471-230X/11/85/prepub
